# Mitochondrial Dysfunction Contributes to Hypertensive Target Organ Damage: Lessons from an Animal Model of Human Disease

**DOI:** 10.1155/2016/1067801

**Published:** 2016-08-09

**Authors:** Speranza Rubattu, Rosita Stanzione, Massimo Volpe

**Affiliations:** ^1^IRCCS Neuromed, 86077 Pozzilli, Italy; ^2^Department of Clinical and Molecular Medicine, School of Medicine and Psychology, Sapienza University of Rome, Ospedale S. Andrea, 00189 Rome, Italy

## Abstract

Mechanisms underlying hypertensive target organ damage (TOD) are not completely understood. The pathophysiological role of mitochondrial oxidative stress, resulting from mitochondrial dysfunction, in development of TOD is unclear. The stroke-prone spontaneously hypertensive rat (SHRSP) is a suitable model of human hypertension and of its vascular consequences. Pathogenesis of TOD in SHRSP is multifactorial, being determined by high blood pressure levels, high salt/low potassium diet, and genetic factors. Accumulating evidence points to a key role of mitochondrial dysfunction in increased susceptibility to TOD development of SHRSP. Mitochondrial abnormalities were described in both heart and brain of SHRSP. Pharmacological compounds able to protect mitochondrial function exerted a significant protective effect on TOD development, independently of blood pressure levels. Through our research efforts, we discovered that two genes encoding mitochondrial proteins, one (Ndufc2) involved in OXPHOS complex I assembly and activity and the second one (UCP2) involved in clearance of mitochondrial ROS, are responsible, when dysregulated, for vascular damage in SHRSP. The suitability of SHRSP as a model of human disease represents a promising background for future translation of the experimental findings to human hypertension. Novel therapeutic strategies toward mitochondrial molecular targets may become a valuable tool for prevention and treatment of TOD in human hypertension.

## 1. Introduction

It has been clearly understood that the complexity of the etiopathogenetic basis of human hypertension extends also to the etiopathogenesis of the related TOD [1]. An interplay of several environmental and genetic factors, not excluding the epigenetic regulation, modulates susceptibility to TOD in hypertensive patients. In order to achieve an optimal control of the vascular consequences related to high BP, we need to dissect out the single players and to understand the underlying mechanistic pathways. Several efforts have been performed over the last two decades to identify the most relevant determinants of TOD in hypertensive animal models. Among the latter, the SHRSP represents a suitable experimental tool.

The SHRSP was established from the SHR 40 years ago based on its higher susceptibility to develop spontaneous cerebrovascular accidents [2]. It develops a severe form of hypertension and shows a shorter lifespan (about one year), as compared to the stroke-resistant SHR (SHRSR) [3, 4]. One-year follow-up of both strains maintained under regular dietary feeding revealed increased occurrence of renal, cerebrovascular, and cardiac damage in SHRSP as compared to the SHRSR in association with higher BP levels [4]. Interestingly, predisposition to stroke is accelerated in the SHRSP by feeding with a high salt/low potassium dietary regimen (Japanese style diet) and it is preceded by renal damage occurrence [5, 6]. Notably, the SHRSR does not show either renal damage or the stroke phenotype when exposed to the same high salt diet despite BP levels comparable to those of SHRSP, thus indicating a peculiar susceptibility of the SHRSP strain to high salt induced vascular damage [7, 8].

The SHRSP can be considered a suitable animal model for studies of human hypertension since it shares several features in common with the human pathological condition. In particular, high BP levels, dietary salt excess, and reduced potassium intake favour cardiovascular damage development, such as cerebrovascular and renal disease, in both SHRSP and in humans [9].

The type of stroke detected in SHRSP is predominantly ischemic (80%) and to a lesser extent hemorrhagic (20%), similarly to humans. Histopathological studies of the brain reveal most of the key signs of small vessel disease: segmental wall damage, depletion of smooth myocytes, accumulation of fibrous tissue, wall thickening, luminal narrowing, blood brain barrier leakage, and perivascular oedema. These alterations can be summarized as a fibrinoid vascular lesion caused by a degenerative-infiltrative-proliferative disease process which affects short segments of penetrating arterioles in multiple, wide scattered foci [10, 11].

## 2. Pathophysiological Studies of TOD in SHRSP

The pathophysiological evidence obtained over the years in the SHRSP supports a major role of higher BP levels in producing increased susceptibility to TOD. On the other hand, as previously mentioned, additional causative factors appear to be involved, being triggered by high salt/low potassium dietary feeding. Previous studies exploring the mechanistic aspects of increased vascular damage in high salt fed SHRSP found implications of the RAAS abnormalities [5], of increased oxidative stress, as compared to both WKY and SHRSR strains [12–14], of reduced NO bioavailability, the latter being the result of NO scavenging by the increased levels of anion superoxide [15], of increased local and systemic inflammation with accumulation of acute-phase proteins in body fluids [16].

In fact, therapeutic strategies aimed at counteracting the RAAS activation with ACEI and ARB turned out to be more beneficial than beta-blocker and calcium antagonist drugs despite comparable BP lowering effect [17–19]. Interestingly, a high potassium diet improved life survival, independently of BP levels reduction [20]. Moreover, interventional strategies able to counteract the increased rate of oxidative stress and of systemic inflammation were able to prevent the development of TOD and to increase SHRSP survival [19, 21–23]. Increasing vascular NO levels through a gene therapy approach restored endothelial function [24].

Thus, although hypertension remained a major permissive factor, the above-mentioned evidence indicated that hypertensive TOD had a complex multifactorial pathogenesis in this animal model. In particular, the evidence that SHRSP developed stroke whereas SHRSR did not upon the same high salt dietary feeding, despite similar BP levels, prompted us several years ago to search for BP independent contributing factors. In fact, the existence of hypertension independent etiopathogenetic determinants underlying stroke in this animal model was convincingly demonstrated through the genetic approach [7].

## 3. Genetic Studies of TOD in SHRSP

The availability of an inbred genetically homogenous animal model represents a great challenge for the discovery of genes contributing to both hypertension and to the related vascular damage [25]. First of all, a specific genetic background was shown to determine development of hypertension in SHRSP [26–29]. Subsequently, in order to dissect out genes contributing to stroke in hypertension, few research efforts were performed through a genetic linkage analysis approach in F2-hybrid cohorts from either SHRSR/SHRSP or SHRSP/WKY intercrosses [7, 30, 31]. The results of these studies revealed the existence of genes playing a direct contributory role in the stroke phenotype occurrence mapping within different areas (QTL) of the rat genome. Moreover, the existence of genes for renal damage was revealed in SHRSP [32].

Although no specific gene predisposing to either hypertension or its vascular consequences has been precisely identified yet in SHRSP, strong evidence has been collected over the last few years with regard to few candidate genes (mapping within the identified stroke QTLs) and their encoded proteins involved in both stroke and renal damage development.

In this regard, an altered sequence and regulation of the atrial natriuretic peptide gene, mapping at the lod score peak of a QTL on rat chromosome 5, STR2, in the SHRSR/SHRSP F2-hybrid cohort [7], were found in SHRSP, a finding later on translated to humans, as described in detail in previous review articles [35, 36]. Interesting observations were also reported with regard to Stim1 gene and the response to stress in SHRSP [34]. Additional genes that emerged as putative candidate for both brain and renal vascular damage in the SHRSP are shown in Table 1 [33, 37–39].

In the present paper, we will discuss the available evidence related to genes encoding mitochondrial proteins, underlying both stroke and renal damage development in SHRSP, that were discovered within STR1.

## 4. In the Search for Stroke Genes Mapping within the Stroke QTL STR1 in SHRSP

The major finding of the linkage analysis performed in the SHRSR/SHRSP F2-hybrid cohort was the identification of STR1, a QTL included between* Klk* and* Mt1pa* markers, extending for an overall 80 cM length and showing a 6.7 lod score peak in correspondence with the anonymous marker* D1Mit3*. Based on our studies, STR1 contributed to 20% of the overall stroke phenotype variance [7]. Notably, its stroke contributory effect was confirmed by congenic experimentation [8]. In fact, congenic lines were prepared by performing several backcrosses of SHRSP with SHRSR [8]. As a result, the congenic animals carrying the STR1/QTL in the SHRSP configuration within the SHRSR genomic background had increased stroke occurrence, with respect to the SHRSR parental strain of origin. Vice versa, the congenic animals carrying the STR1/QTL in the SHRSR configuration within the SHRSP genomic background showed decreased stroke occurrence as compared to the SHRSP parental strain of origin [8]. These results clearly indicated that the STR1/QTL contained one or more genes directly contributing to stroke predisposition in the animal model. In fact, subsequent studies, addressed to identify the potential genetic determinants of stroke contained within the STR1/QTL, revealed novel mechanisms contributing to vascular damage.

In particular, the mitochondrial dysfunction was discovered as a crucial mechanism underlying stroke development in SHRSP.

## 5. Mitochondria as a Source of ROS and of Vascular Damage in Hypertension

ROS behave as an important intracellular and intercellular second messenger that modulate several downstream signaling molecules leading to vascular smooth muscle cell growth and migration, expression of proinflammatory mediators, and remodeling of extracellular matrix [40]. In addition, oxidative stress increases intracellular free calcium concentration, a major determinant of vascular reactivity [41]. The most relevant sources of ROS with respect to vascular disease and hypertension are NADPH oxidase; NOS; xanthine oxidase; and mitochondria. A feedforward interplay exists between these ROS sources [42]. For instance, activation of NADPH oxidases by angiotensin II increases mitochondrial superoxide production [43].

Mitochondria represent a major source of energy for cells and their function is fundamental for life of all organisms [44]. They mediate oxidative phosphorylation via electrons transfer through multimeric complexes to produce ATP. Electrons are donated from NADH to complex I and then to other components of the chain in order to reduce molecular oxygen (O_2_) to form H_2_O. Flow of electrons through the OXPHOS chain is accompanied by pumping of protons across the mitochondrial inner membrane, thus creating a transmembrane electrochemical gradient. Complex I, known as NADH:ubiquinone oxidoreductase, is the largest of the five OXPHOS complexes and it is composed of 45 subunits (seven encoded by mitochondrial DNA and 38 encoded by nuclear DNA). Complex I leaks electrons in the intermembrane space to generate mitochondrial anion superoxide (O_2_^−^). The reentry of protons into the matrix is used by complex V to synthetize ATP from ADP [44]. Thus, complex I contributes to about 40% of the proton motive force that drives ATP synthesis by ATP synthase and, along with complex III, is the major source of O_2_^−^ production [44].

Under physiological condition, ROS are produced as a byproduct of the respiratory chain due to 1-electron reduction of O_2_ to O_2_^−^ [44]. Mitochondria are the major source of cellular ROS and they also contain antioxidant mechanisms such as mitochondrial matrix enzyme manganese superoxide dismutase, glutathione peroxidase, and peroxiredoxins 3 and 5 [45]. Accumulation of ROS, as a result of an imbalance between production and clearance, leads to damage of mitochondrial proteins of lipids and of DNA with consequent alteration of the mitochondrial function and further increase of ROS. Moreover, mitochondrial superoxide stimulates cytoplasmic NADPH oxidase activity in endothelial cells and therefore further contributes to development of endothelial oxidative stress [46].

It is becoming more and more clear that mitochondrial dysfunction causing excessive ROS production may contribute to the pathogenesis of common cardiovascular diseases [47]. Notably, mitochondria may be involved in the genesis of hypertension and hypertension itself may promote mitochondrial dysfunction in the brain, heart, vasculature, and kidneys [48].

The mechanisms underlying the increased mitochondrial oxidative stress which has been described in the cardiovascular system of several models of hypertension are multiple [48]. Through a crosstalk interaction between NADPH oxidases and mitochondria, the stimulation of vascular NADPH oxidases, for instance, by angiotensin II, increases mitochondrial oxidative stress [48]. Notably, excess salt, through ROS produced by angiotensin II-activated NADPH oxidase, caused cerebral neuronal apoptosis and inflammation as well as stroke in SHRSP [49]. A mechanosensitive blood pressure-induced mitochondrial ROS production has been shown to exacerbate vascular oxidative stress in hypertension [50]. In addition to pressure, a shear stress-induced mitochondrial ROS is considered an important mechanism at the endothelial level [48, 51]. Along with the above-mentioned evidence, an imbalance between ROS production and the mitochondrial antioxidant systems contributes to increased oxidative stress in hypertension [48]. Increased mitochondrial oxidative stress may sustain hypertension as suggested by evidence that activation of the RAAS stimulates mitochondrial redox signaling pathways and leads to augmented neuronal firing that exerts a key role in the central regulation of BP [52].

Mitochondrial dysfunction may also contribute to promoting hypertensive TOD. In this regard, an assembly defect in complex I and in complex V of the OXPHOS in SHR brain mitochondria, causing a decreased ATP production and cell energy deficiency, was previously described [53]. Evidence of cardiac mitochondrial damage was documented in experimental models of hypertension, including the SHR [54, 55]. Mitochondrial abnormalities were described in the heart of SHR [56] and in the kidneys of both renovascular and essential hypertensive animal models [57, 58].

The SHRSP provides key insights into the contribution of mitochondrial dysfunction in the pathogenesis of vascular damage associated with hypertension. This concept was hypothesized in SHRSP for the first time several years ago, based on evidence of a significant increase of mitochondria in cardiomyocytes of lacidipine treated SHRSP [59]. The presence of alterations in mitochondrial DNA and enzyme activities was described in the hypertrophied heart of SHRSP [60]. Mitochondrial abnormalities were also related to the brain damage following middle cerebral artery occlusion in SHRSP [61].

Of note, treatment with S 35171, a compound able to protect mitochondrial function by increasing the inner antioxidant systems, was shown to delay and even to prevent brain damage in high salt fed SHRSP, as a result of protection from systemic processes linked to mitochondria and involved in brain damage [62]. Notably, this compound had only a modest, not significant effect on BP levels [62]. Furthermore, a therapeutic strategy targeted at the mitochondria oxidative stress through mitoQ10 administration (either alone or added to losartan) was shown to exert beneficial effects on BP levels reduction and, particularly, on protection from the associated vascular and cardiac damage [63, 64]. Interestingly, mitoQ10, which is specifically directed toward mitochondrial oxidative stress, attenuated the hypertension development but it did not prevent it. Parallel in vitro studies showed dose-dependent inhibition of hypertrophy in cardiomyocytes exposed to mitoQ10 indicating a direct prohypertrophic effect of mitochondrial ROS [63].

Moreover, the significant protective effect described with fenofibrate on both renal and cerebral damage in salt loaded SHRSP [65] may be mediated by its PPAR*α* agonist action and the consequent stimulation of UCP2 expression levels [66].

## 6. Ndufc2 as a Stroke Susceptibility Gene within STR1

Through a microarray differential expression analysis, including all sequences mapping inside STR1, we discovered the gene encoding the mitochondrial protein Ndufc2 (a subunit of the OXPHOS complex I, encoded by nuclear DNA) as a sequence significantly downregulated by high salt diet only in the brain of SHRSP as compared to the SHRSR [13]. This evidence associated with signs of mitochondrial dysfunction, such as reduced ATP and increased ROS levels. Furthermore, we discovered that the* Ndufc2* silencing in vascular cells led to a significant impairment of complex I assembly, to a reduction of mitochondrial membrane potential and of ATP synthesis, with consequent marked accumulation of ROS, increased inflammation, increased cell necrosis, and reduced cell viability [13]. In vivo, a heterozygous* Ndufc2*-ko rat model, obtained from the parental SHRSR strain, showed a deficient complex I assembly and, more importantly, once fed with the Japanese style diet, developed renal damage followed by stroke occurrence, similarly to what occurs in SHRSP [13]. No differences in BP levels were detected in the* Ndufc2*-ko model as compared to its strain of origin under the same high salt diet, thus underscoring a direct contributory role of mitochondrial dysfunction in the development of vascular damage. As a consequence of single gene effect, the percentage of stroke events did not go above 40%. The identification of a marker of the human gene, associated with a significant NDUFC2 defective expression in carriers and with early-onset ischemic stroke occurrence [13], supports the hypothesis that reduced NDUFC2 expression could contribute to increased stroke susceptibility also in humans.

**Figure 1:**
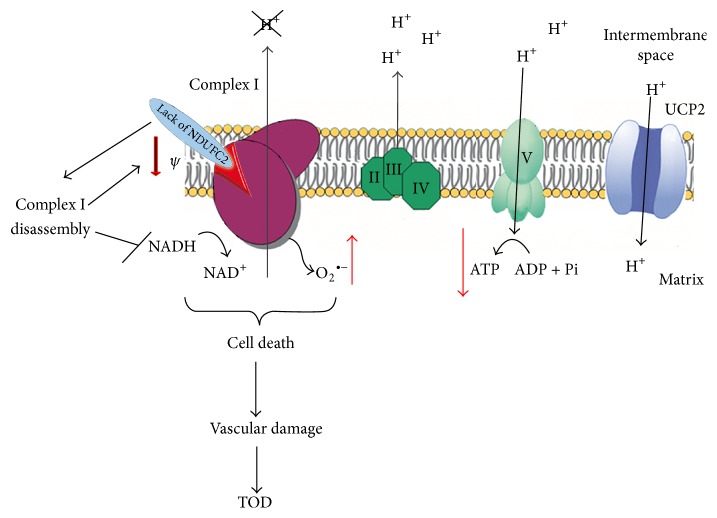
Schematic representation of the electron transport chain OXPHOS within the inner mitochondrial membrane. Complex I is highlighted to underscore its major relevance as a determinant of excessive mitochondrial ROS production when it becomes dysfunctional. In particular, lack of Ndufc2 subunit of complex I is highlighted to indicate that it leads to disassembly and dysfunction of the complex. As a result, NADH cannot be converted to NAD^+^, with consequent reduction of the flux of protons into the matrix, significant decrease of mitochondrial membrane potential, increase of anion superoxide, and reduction of ATP synthesis. The resulting cellular and tissue damage can contribute to target organ damage development in hypertension.

Function of the Ndufc2 is starting to be understood [67]. The observations obtained in the SHRSP reveal a key role of Ndufc2 in complex I assembly, a critical step for an appropriate complex I activity and for the whole OXPHOS function (Figure 1). Of note, these findings parallel and reinforce previous data obtained in type 2 diabetes mellitus [68]. Moreover, NDUFC2 appears downregulated in skeletal muscle cells of subjects affected by insulin resistance [69] and it is associated with insulin secretion in vivo [70].* Ndufc2* expression is induced under Bendavia treatment, a novel molecule able to counteract mitochondrial dysfunction, in the noninfarcted border zone within the rat heart, underscoring a key role of a regular complex I activity in ischemic heart disease [71]. NDUFC2 may also represent a novel oncogene involved in breast cancer and intestinal adenocarcinoma, predicting poor prognosis [72, 73]. Interestingly, an epigenetic regulation may contribute to modulating Ndufc2 expression. In fact, this gene has been described, but not yet experimentally proven, as a predicted target of miRNA34a. The latter is a small noncoding RNA exerting a significant impact on mitochondrial function and on blood brain barrier integrity [74].

Whereas the above-mentioned evidence highlights the role of NDUFC2 as a novel gene for human diseases, the results obtained in SHRSP bring up to our attention for the first time the importance of an adequate mitochondrial function, through a regular complex I assembly and activity, in order to maintain the vascular health status and to avoid TOD development in hypertension. Of note, complex I mediated production of mitochondrial O_2_^−^ has been implicated so far in ischemic preconditioning, in aging and, only indirectly, in hypertension [75]. Deficiency of complex I causes neurotoxicity [76].

## 7. UCP2 as a Suitable Candidate Gene for Renal Damage in SHRSP

Identification of UCP2 mapping nearby the load score peak of STR1 revealed unexpected interesting implications for the renal injury phenotype of SHRSP. UCP2, belonging to the uncoupling proteins family, is located within the inner mitochondrial membrane where it carries negative ions (H^+^) and it dissipates the mitochondrial proton gradient that contributes to anion superoxide formation by reverse electron flow from complex II to complex I [77]. Its dysregulation has been associated with either high salt or obesity related vascular dysfunction [78–80]. On the other hand, UCP2 overexpression is able to restore a normal vascular function [81, 82]. It has been suggested that an impairment of the protective role of UCP2 may also contribute to hypertension development [83]. Interestingly, mice overexpressing UCP2 show a longer lifespan [84].

We discovered a differential regulation of UCP2 expression levels, upon the stroke-permissive high salt diet, in kidneys of SHRSP versus SHRSR with evidence of reduced expression in stroke-prone and increased expression in stroke-resistant rats [14]. We also provided evidence that knocking out UCP2 gene in renal cells led to increased oxidative stress, reduced ATP synthesis, and increased cell necrosis [14]. Our experimental findings are consistent with previous evidence showing an involvement of UCP2 in human kidney disease [85–87].

Interestingly, the stimulation of renal UCP2 expression, by administration of a vegetable extract obtained from* Brassica oleracea* sprouts, was able to counteract the high salt diet induced renal damage of SHRSP, independently of BP levels [88]. The key role of UCP2 stimulation in mediating the positive effects of* Brassica oleracea* sprouts extract was reinforced by evidence that parallel blockade with a selective inhibitor of PPAR*α*, a family of nuclear transcriptional factor receptors that regulate several genes including UCP2 [66], did not allow any further protection from renal damage in high salt fed SHRSP [88].

A similar striking differential modulation, that is suppression of UCP2 gene and protein expression, was found in relation to both hypertension and aging in the brain, heart, and kidneys of SHRSP but not of SHRSR [4]. Thus, based on the reported evidence, high salt diet, high blood pressure levels, and aging are able to turn off tissue UCP2 gene expression only in SHRSP through still unexplained mechanisms. In this regard, an epigenetic regulation may be involved, as suggested by evidence obtained in relation to the kidney injury of this model [13].

**Figure 2:**
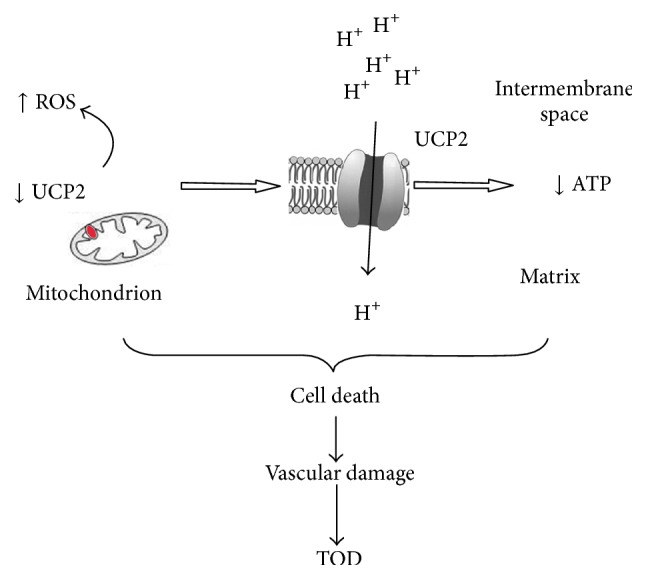
Schematic representation of the effects of UCP2 downregulation within the inner mitochondrial membrane. As a consequence of the reduced UCP2 function, a lower number of protons are shifted into the matrix with a consequent increase of reactive oxygen species and dysfunction of the whole OXPHOS with decreased ATP synthesis and cellular damage.

Consistent with the critical role of UCP2 in clearance of ROS, whenever UCP2 expression is decreased levels of both oxidative stress and inflammation are remarkably increased, and levels of ATP are decreased with consequent cellular and tissue damage (Figure 2). Restoring UCP2 expression levels, as observed under the* Brassica oleracea* sprouts extract, appears a critical step to prevent TOD development in hypertension [88]. Altogether, these data indicate that UCP2 plays a role in the setting of hypertensive TOD.

Of note, the beneficial cardiovascular effects of vegetable compounds, including* Brassica oleracea*, were also reported in humans [89], thus underscoring their potential useful role as an adjuvant to conventional cardiovascular therapies. The improvement of mitochondrial function by the vegetables administration is often mediated through UCP2 upregulation [89].

Overall, the results of our experimental research in the SHRSP strongly support the hypothesis that mitochondrial dysfunction may be an important contributory pathogenetic mechanism underlying hypertensive vascular damage, independently of BP levels, and that the SHRSP is a suitable animal model of mitochondrial oxidative stress-dependent hypertensive TOD. In fact, through the discovery of two mitochondrial proteins as key determinants of both cerebral and renal damage, a cause-effect relationship between mitochondrial dysfunction and hypertensive vascular damage was revealed in a clear manner in the SHRSP. Of note, apart from the two genes discussed herein, some of those listed in Table 1 may also contribute to mitochondrial dysfunction and to vascular damage in this animal model. Among all, the mitochondrial HMG-CoA synthase plays an antioxidant protective role in the kidneys [33], similarly to UCP2.

Notably, a gene-environment interaction appears critical to unmask the inherited mitochondrial dysfunction of SHRSP. The precise mechanisms by which salt excess interferes with mitochondrial function still need to be clarified. The role of an epigenetic regulation, as discussed for both Ndufc2 and UCP2, cannot be excluded.

Apart from the positive lessons learned from this animal model, some drawbacks should also be outlined. First of all, the complex nature of human hypertensive vascular damage certainly relies on additional genetic factors that remained undiscovered in SHRSP due either to technical limitations of the methodological approaches or to limitations inherent in the model. For instance, no information could be obtained through the use of this model on the role of mitochondrial DNA variants on oxidative vascular damage in hypertension. In this regard, it is known that mitochondrial DNA plays a role in distinct pathological contexts [90]. Its pathogenic role in hypertensive TOD still needs to be clarified. Moreover, by specifically studying mitochondrial dysfunction in a peculiar model of stroke and renal damage associated with hypertension, other relevant pathogenetic contributions of this molecular mechanism in cardiovascular diseases (ischemic heart disease, left ventricular hypertrophy, and metabolic syndrome) are missed.

## 8. Expected Relevance of the Experimental Findings for Human Hypertensive Disease

The consequences of a dysregulated mitochondrial function obviously apply to each living organism through the cell and tissue damage consequent to the increased oxidative stress and reduced ATP production. In fact, mitochondrial dysfunction has been already established as an important pathogenetic determinant of common neurodegenerative diseases in humans [91].

In contrast, current knowledge on the role of mitochondrial dysfunction in human hypertensive vascular damage is still very scarce. The animal model reveals exciting new results in this regard by highlighting the role of a complex I subunit and of UCP2 into increased renal and cerebrovascular damage. We expect to see a translation of the evidence obtained in SHRSP to the human disease, since several findings reported in this model often anticipated those encountered later on in the human pathological context. For instance, we were able to translate to humans the information provided by SHRSP about the role of the atrial natriuretic peptide gene as a determinant of stroke [35, 36]. The current preliminary translation to human ischemic stroke of the results obtained in SHRSP with regard to the contributory role of* Ndufc2* looks promising [13]. It suggests that a defective complex I may play a pathogenic role also in human stroke. Larger genetic human studies will better assess this evidence.

Furthermore, evidence that UCP2 is involved in human chronic kidney disease [92, 93], including that of hypertensive origin [85], reinforces the implications of the experimental findings obtained in SHRSP and underscores an important role of this mitochondrial protein in human pathology as well.

Future studies will better define the true pathogenetic relevance of mitochondrial dysfunction in both animal models and human hypertensive vascular disease. It cannot be excluded that an improvement of mitochondrial dysfunction may represent a mechanism by which available antihypertensive drugs can modulate vascular oxidative stress and exert vascular protection beyond their BP lowering effect. In addition, novel targeted strategies, designed toward mitochondria dependent pathogenic mechanisms [94], may reveal a useful therapeutic tool through their ability to improve mitochondrial function and therefore to protect patients from TOD development in hypertension. In this regard, the SHRSP represents a precious tool to gain insights into the mechanisms of cardiovascular actions of both old and novel antihypertensive pharmacological agents.

## Figures and Tables

**Table 1:** Genes identified in SHRSP in relation to increased susceptibility to vascular damage.

Gene name	Experimental context	Reference
Ndufc2	Salt loading (brain)	[13]
UCP2	Salt loading (kidneys)	[14]
Mit HMG-CoA synthase	Standard chow (kidneys)	[33]
Nrf2	Standard chow (arteries and VSMC)	[12]
Agt	Standard chow (adrenal glands, kidneys, brain)	[38
Stim1	Exaggerated sympathetic response to stress	[34]
Atrial natriureticpeptide	Salt loading (brain)	[35, 36]
MMP14	Gene expression analysis of brain SVD	[37]
Gnai1	Gene expression analysis of brain SVD	[39
Vasopressin	Gene expression analysis of brain SVD	[37]
Albumin	Gene expression analysis of brain SVD	[37]
NO receptor	Gene expression analysis of brain SVD	[37]
Gucy1a3	Gene expression analysis of brain SVD	[37]
Rps9	Gene expression analysis of brain SVD	[37]
Edg1	Salt loading (kidneys)	[38]
Vcam1	Salt loading (kidneys)	[38]

Mit HMG-CoA: mitochondrial 3-hydroxy-3-methylglutaryl-coenzyme A; Gnai1: guanine nucleotide binding protein alpha inhibitor 1; Nrf2: nuclear factor erythroid 2-related factor; Agt: angiotensinogen; Stim1: stromal interaction molecule 1; NO: nitric oxide; MMP14: metalloproteinase 14; Rps9: ribosomal protein S9; Gucy1a3: guanylate cyclase 1, soluble, alpha 3; Edg1: endothelial differentiation gene receptor 1; Vcam1: vascular cell adhesion molecule 1; SVD: small vessel disease.

## Abbreviations

ACEI: Angiotensin converting enzyme inhibitor
ARB: Angiotensin receptor blocker
ADP: Adenosine diphosphate
ATP: Adenosine triphosphate
BP: Blood pressure
NADPH: Nicotinamide adenine dinucleotide phosphate
NDUFC2: NADH:ubiquinone oxidoreductase subunit C2
NO: Nitric oxide
NOS: Nitric oxide synthase
OXPHOS: Oxidative phosphorylation
PPAR*α*: Peroxisome proliferator activated receptor alpha
QTL: Quantitative trait locus
RAAS: Renin-angiotensin-aldosterone system
ROS: Reactive oxygen species
SHRSP: Stroke-prone spontaneously hypertensive rat
SHRSR: Stroke-resistant spontaneously hypertensive rat
STR1: QTL for stroke on rat chromosome 1 in SHRSP
STR2: QTL for stroke on rat chromosome 5 in SHRSP
TOD: Target organ damage
UCP2: Uncoupling protein 2
WKY: Wistar Kyoto rat, normotensive.

## References

[1] Churchill P. C., Churchill M. C., Bidani A. K. (1997). Genetic susceptibility to hypertension-induced renal damage in the rat: evidence based on kidney-specific genome transfer. *Journal of Clinical Investigation*.

[2] Okamoto K., Yamori Y., Nagaoka A. (1974). Establishment of the stroke prone spontaneously hypertensive rat (SHR). *Circulation Research*.

[3] Sarwar G., Ratnayake W. M. N., Mueller R. (1999). Longevity of the stroke-prone hypertensive rats is influenced by the source and amount of dietary protein. *Nutrition Research*.

[4] Rubattu S., Bianchi F., Busceti C. L. (2015). Differential modulation of AMPK/PPAR*α*/UCP2 axis in relation to hypertension and aging in the brain, kidneys and heart of two closely related spontaneously hypertensive rat strains. *Oncotarget*.

[5] Volpe M., Camargo M. J. F., Mueller F. B. (1990). Relation of plasma renin to end organ damage and to protection of K+ feeding in stroke-prone hypertensive rats. *Hypertension*.

[6] Schreiber S., Bueche C. Z., Garz C. (2011). Kidney pathology precedes and predicts the pathological cascade of cerebrovascular lesions in stroke prone rats. *PLoS ONE*.

[7] Rubattu S., Volpe M., Kreutz R., Ganten U., Ganten D., Lindpaintner K. (1996). Chromosomal mapping of quantitative trait loci contributing to stroke in a rat model of complex human disease. *Nature Genetics*.

[8] Rubattu S., Hubner N., Ganten U. (2006). Reciprocal congenic lines for a major stroke QTL on rat chromosome 1. *Physiological Genomics*.

[9] Shay C. M., Gooding H. S., Murillo R., Foraker R. (2015). Understanding and improving cardiovascular health: an update on the American Heart Association's concept of cardiovascular health. *Progress in Cardiovascular Diseases*.

[10] Hainsworth A. H., Markus H. S. (2008). Do in vivo experimental models reflect human cerebral small vessel disease? A systematic review. *Journal of Cerebral Blood Flow and Metabolism*.

[11] Fredriksson K., Nordborg C., Kalimo H., Olsson Y., Johansson B. B. (1988). Cerebral microangiopathy in stroke-prone spontaneously hypertensive rats. An immunohistochemical and ultrastructural study. *Acta Neuropathologica*.

[12] Lopes R. A., Neves K. B., Tostes R. C., Montezano A. C., Touyz R. M. (2015). Downregulation of nuclear factor erythroid 2-related factor and associated antioxidant genes contributes to redox-sensitive vascular dysfunction in hypertension. *Hypertension*.

[13] Rubattu S., Di Castro S., Schulz H. (2016). Ndufc2 gene inhibition is associated with mitochondrial dysfunction and increased stroke susceptibility in an animal model of complex human disease. *Journal of the American Heart Association*.

[14] Di Castro S., Scarpino S., Marchitti S. (2013). Differential modulation of uncoupling protein 2 in kidneys of stroke-prone spontaneously hypertensive rats under high-salt/low-potassium diet. *Hypertension*.

[15] Kerr S., Brosnan M. J., McIntyre M., Reid J. L., Dominiczak A. F., Hamilton C. A. (1999). Superoxide anion production is increased in a model of genetic hypertension: role of the endothelium. *Hypertension*.

[16] Sironi L., Tremoli E., Miller I. (2001). Acute-phase proteins before cerebral ischemia in stroke-prone rats: identification by proteomics. *Stroke*.

[17] Chillon J.-M., Baumbach G. L. (1999). Effects of an angiotensin-converting enzyme inhibitor and a *β*-blocker on cerebral arterioles in rats. *Hypertension*.

[18] Kim-Mitsuyama S., Yamamoto E., Tanaka T. (2005). Critical role of angiotensin II in excess salt-induced brain oxidative stress of stroke-prone spontaneously hypertensive rats. *Stroke*.

[19] Sironi L., Gelosa P., Guerrini U. (2004). Anti-inflammatory effects of AT1 receptor blockade provide end-organ protection in stroke-prone rats independently from blood pressure fall. *Journal of Pharmacology and Experimental Therapeutics*.

[20] Tobian L., Lange J. M., Johnson M. A. (1986). High-K diets reduce brain haemorrhage and infarcts, death rate and mesenteric arteriolar hypertrophy in stroke-prone spontaneously hypertensive rats. *Journal of Hypertension*.

[21] Nagotani S., Hayashi T., Sato K. (2005). Reduction of cerebral infarction in stroke-prone spontaneously hypertensive rats by statins associated with amelioration of oxidative stress. *Stroke*.

[22] Sironi L., Gianazza E., Gelosa P. (2005). Rosuvastatin, but not simvastatin, provides end-organ protection in stroke-prone rats by antiinflammatory effects. *Arteriosclerosis, Thrombosis, and Vascular Biology*.

[23] Kawashima S., Yamashita T., Miwa Y. (2003). HMG-CoA reductase inhibitor has protective effects against stroke events in stroke-prone spontaneously hypertensive rats. *Stroke*.

[24] Alexander M. Y., Brosnan M. J., Hamilton C. A. (1999). Gene transfer of endothelial nitric oxide synthase improves nitric oxide dependent endothelial function in a hypertensive rat model. *Cardiovascular Research*.

[25] Nabika T., Ohara H., Kato N., Isomura M. (2012). The stroke-prone spontaneously hypertensive rat: still a useful model for post-GWAS genetic studies?. *Hypertension Research*.

[26] Jacob J. J., Lindpaintner K., Lincoln S. E. (1991). Genetic mapping of a gene causing hypertension in the stroke-prone spontaneously hypertensive rat. *Cell*.

[27] Kreutz R., Hübner N., James M. R. (1995). Dissection of a quantitative trait locus for genetic hypertension on rat chromosome 10. *Proceedings of the National Academy of Sciences of the United States of America*.

[28] Koh-Tan H. H. C., McBride M. W., McClure J. D. (2013). Interaction between chromosome 2 and 3 regulates pulse pressure in the stroke-prone spontaneously hypertensive rat. *Hypertension*.

[29] Kato N., Nabika T., Liang Y.-Q. (2003). Isolation of a chromosome 1 region affecting blood pressure and vascular disease traits in the stroke-prone rat model. *Hypertension*.

[30] Gandolgor T.-A., Ohara H., Cui Z.-H. (2013). Two genomic regions of chromosomes 1 and 18 explain most of the stroke susceptibility under salt loading in stroke-prone spontaneously hypertensive Rat/Izm. *Hypertension*.

[31] Jeffs B., Clark J. S., Anderson N. H. (1997). Sensitivity to cerebral ischaemic insult in a rat model of stroke is determined by a single genetic locus. *Nature Genetics*.

[32] Gigante B., Rubattu S., Stanzione R. (2003). Contribution of genetic factors to renal lesions in the stroke-prone spontaneously hypertensive rat. *Hypertension*.

[33] Yi W., Fu P., Fan Z. (2010). Mitochondrial HMG-CoA synthase partially contributes to antioxidant protection in the kidney of stroke-prone spontaneously hypertensive rats. *Nutrition*.

[34] Ferdaus M. Z., Xiao B., Ohara H. (2014). Identification of Stim1 as a candidate gene for exaggerated sympathetic response to stress in the stroke-prone spontaneously hypertensive rat. *PLoS ONE*.

[35] Volpe M., Rubattu S., Burnett J. (2014). Natriuretic peptides in cardiovascular diseases: current use and perspectives. *European Heart Journal*.

[36] Rubattu S., Sciarretta S., Volpe M. (2014). Atrial natriuretic peptide gene variants and circulating levels: implications in cardiovascular diseases. *Clinical Science*.

[37] Bailey E. L., Mcbride M. W., Beattie W. (2014). Differential gene expression in multiple neurological, inflammatory and connective tissue pathways in a spontaneous model of human small vessel stroke. *Neuropathology and Applied Neurobiology*.

[38] Graham D., McBride M. W., Gaasenbeek M. (2007). Candidate genes that determine response to salt in the stroke-prone spontaneously hypertensive rat: congenic analysis. *Hypertension*.

[39] Yamamoto H., Okuzaki D., Yamanishi K. (2015). Genetic analysis of genes causing hypertension and stroke in spontaneously hypertensive rats. *International Journal of Molecular Medicine*.

[40] Rubattu S., Pagliaro B., Pierelli G. (2015). Pathogenesis of target organ damage in hypertension: role of mitochondrial oxidative stress. *International Journal of Molecular Sciences*.

[41] Lee M. Y., Griendling K. K. (2008). Redox signaling, vascular function, and hypertension. *Antioxidants & Redox Signaling*.

[42] Schulz E., Wenzel P., Münzel T., Daiber A. (2014). Mitochondrial redox signaling: interaction of mitochondrial reactive oxygen species with other sources of oxidative stress. *Antioxidants and Redox Signaling*.

[43] Doughan A. K., Harrison D. G., Dikalov S. I. (2008). Molecular mechanisms of angiotensin II-mediated mitochondrial dysfunction. Linking mitochondrial oxidative damage and vascular endothelial dysfunction. *Circulation Research*.

[44] Turrens J. F. (2003). Mitochondrial formation of reactive oxygen species. *Journal of Physiology*.

[45] Andreyev A. Y., Kushnareva Y. E., Starkov A. A. (2005). Mitochondrial metabolism of reactive oxygen species. *Biochemistry*.

[46] Nazarewicz R. R., Dikalova A. E., Bikineyeva A., Dikalov S. I. (2013). Nox2 as a potential target of mitochondrial superoxide and its role in endothelial oxidative stress. *American Journal of Physiology—Heart and Circulatory Physiology*.

[47] Yu E., Mercer J., Bennett M. (2012). Mitochondria in vascular disease. *Cardiovascular Research*.

[48] Dikalov S. I., Ungvari Z. (2013). Role of mitochondrial oxidative stress in hypertension. *American Journal of Physiology—Heart and Circulatory Physiology*.

[49] Yamamoto E., Tamamaki N., Nakamura T. (2008). Excess salt causes cerebral neuronal apoptosis and inflammation in stroke-prone hypertensive rats through angiotensin II-induced NADPH oxidase activation. *Stroke*.

[50] Ichimura H., Parthasarathi K., Quadri S., Issekutz A. C., Bhattacharya J. (2003). Mechano-oxidative coupling by mitochondria induces proinflammatory responses in lung venular capillaries. *Journal of Clinical Investigation*.

[51] Liu Y., Zhao H., Li H., Kalyanaraman B., Nicolosi A. C., Gutterman D. D. (2003). Mitochondrial sources of H_2_O_2_ generation play a key role in flow-mediated dilation in human coronary resistance arteries. *Circulation Research*.

[52] Case A. J., Li S., Basu U., Tian J., Zimmerman M. C. (2013). Mitochondrial-localized NADPH oxidase 4 is a source of superoxide in angiotensin II-stimulated neurons. *American Journal of Physiology—Heart and Circulatory Physiology*.

[53] Lopez-Campistrous A., Hao L., Xiang W. (2008). Mitochondrial dysfunction in the hypertensive rat brain: respiratory complexes exhibit assembly defects in hypertension. *Hypertension*.

[54] Tang Y., Mi C., Liu J., Gao F., Long J. (2014). Compromised mitochondrial remodeling in compensatory hypertrophied myocardium of spontaneously hypertensive rat. *Cardiovascular Pathology*.

[55] Eirin A., Lerman A., Lerman L. O. (2014). Mitochondrial injury and dysfunction in hypertension-induced cardiac damage. *European Heart Journal*.

[56] Rodrigues J. Q. D., Da Silva E. D., De Magalhães Galvão K. (2014). Differential regulation of atrial contraction by P1 and P2 purinoceptors in normotensive and spontaneously hypertensive rats. *Hypertension Research*.

[57] De Cavanagh E. M. V., Toblli J. E., Ferder L., Piotrkowski B., Stella I., Inserra F. (2006). Renal mitochondrial dysfunction in spontaneously hypertensive rats is attenuated by losartan but not by amlodipine. *American Journal of Physiology—Regulatory Integrative and Comparative Physiology*.

[58] Eirin A., Li Z., Zhang X. (2012). A mitochondrial permeability transition pore inhibitor improves renal outcomes after revascularization in experimental atherosclerotic renal artery stenosis. *Hypertension*.

[59] Cristofori P., Sbarbati A., Accordini C., Terron A., Micheli D. (1994). Protective action of lacidipine in cardiac hypertrophy of the spontaneously hypertensive stroke-prone rat: an ultrastructural study. *Journal of Submicroscopic Cytology and Pathology*.

[60] Tokoro T., Ito H., Suzuki T. (1996). Alterations in mitochondrial DNA and enzyme activities in hypertrophied myocardium of stroke-prone SHRS. *Clinical and Experimental Hypertension*.

[61] Onoue S., Kumon Y., Igase K., Ohnishi T., Sakanaka M. (2005). Growth arrest and DNA damage-inducible gene 153 increases transiently in the thalamus following focal cerebral infarction. *Molecular Brain Research*.

[62] Gelosa P., Banfi C., Brioschi M. (2009). S 35171 exerts protective effects in spontaneously hypertensive stroke-prone rats by preserving mitochondrial function. *European Journal of Pharmacology*.

[63] Graham D., Huynh N. N., Hamilton C. A. (2009). Mitochondria-targeted antioxidant MitoQ_10_ improves endothelial function and attenuates cardiac hypertrophy. *Hypertension*.

[64] McLachlan J., Beattie E., Murphy M. P. (2014). Combined therapeutic benefit of mitochondria-targeted antioxidant, MitoQ10, and angiotensin receptor blocker, losartan, on cardiovascular function. *Journal of Hypertension*.

[65] Gelosa P., Banfi C., Gianella A. (2010). Peroxisome proliferator-activated receptor *α* agonism prevents renal damage and the oxidative stress and inflammatory processes affecting the brains of stroke-prone rats. *The Journal of Pharmacology and Experimental Therapeutics*.

[66] Tomizawa A., Hattori Y., Inoue T., Hattori S., Kasai K. (2011). Fenofibrate suppresses microvascular inflammation and apoptosis through adenosine monophosphate-activated protein kinase activation. *Metabolism: Clinical and Experimental*.

[67] Mimaki M., Wang X., McKenzie M., Thorburn D. R., Ryan M. T. (2012). Understanding mitochondrial complex I assembly in health and disease. *Biochimica et Biophysica Acta (BBA)—Bioenergetics*.

[68] Gershoni M., Levin L., Ovadia O. (2014). Disrupting mitochondrial-nuclear coevolution affects OXPHOS complex I integrity and impacts human health. *Genome Biology and Evolution*.

[69] Dekker Nitert M., Dayeh T., Volkov P. (2012). Impact of an exercise intervention on DNA methylation in skeletal muscle from first-degree relatives of patients with type 2 diabetes. *Diabetes*.

[70] Olsson A. H., Rönn T., Ladenvall C. (2011). Two common genetic variants near nuclear-encoded OXPHOS genes are associated with insulin secretion in vivo. *European Journal of Endocrinology*.

[71] Shi J., Dai W., Hale S. L. (2015). Bendavia restores mitochondrial energy metabolism gene expression and suppresses cardiac fibrosis in the border zone of the infarcted heart. *Life Sciences*.

[72] Chin S. F., Teschendorff A. E., Marioni J. C. (2007). High-resolution aCGH and expression profiling identifies a novel genomic subtype of ER negative breast cancer. *Genome Biology*.

[73] Igci Y. Z., Bozgeyik E., Borazan E. (2015). Expression profiling of SCN8A and NDUFC2 genes in colorectal carcinoma. *Experimental Oncology*.

[74] Bukeirat M., Sarkar S. N., Hu H., Quintana D. D., Simpkins J. W., Ren X. (2016). MiR-34a regulates blood–brain barrier permeability and mitochondrial function by targeting cytochrome c. *Journal of Cerebral Blood Flow & Metabolism*.

[75] Huang L., Jin X., Xia L. (2016). Characterization of mitochondrial NADH dehydrogenase 1*α* subcomplex 10 variants in cardiac muscles from normal Wistar rats and spontaneously hypertensive rats: implications in the pathogenesis of hypertension. *Molecular Medicine Reports*.

[76] Koene S., Willems P. H. G. M., Roestenberg P., Koopman W. J. H., Smeitink J. A. M. (2011). Mouse models for nuclear DNA-encoded mitochondrial complex I deficiency. *Journal of Inherited Metabolic Disease*.

[77] Mattiasson G., Sullivan P. G. (2006). The emerging functions of UCP2 in health, disease, and therapeutics. *Antioxidants and Redox Signaling*.

[78] Ma S., Ma L., Yang D. (2010). Uncoupling protein 2 ablation exacerbates high-salt intake-induced vascular dysfunction. *American Journal of Hypertension*.

[79] Ma S., Zhang Y., Wang Q. (2014). Ablation of uncoupling protein 2 exacerbates salt-induced cardiovascular and renal remodeling associated with enhanced oxidative stress. *International Journal of Cardiology*.

[80] Moukdar F., Robidoux J., Lyght O., Pi J., Daniel K. W., Collins S. (2009). Reduced antioxidant capacity and diet-induced atherosclerosis in uncoupling protein-2-deficient mice. *Journal of Lipid Research*.

[81] Tian X. Y., Wong W. T., Xu A. (2012). Uncoupling protein-2 protects endothelial function in diet-induced obese mice. *Circulation Research*.

[82] Ma S., Wang Q., Zhang Y. (2014). Transgenic overexpression of uncoupling protein 2 attenuates salt-induced vascular dysfunction by inhibition of oxidative stress. *American Journal of Hypertension*.

[83] Montez P., Vázquez-Medina J. P., Rodríguez R. (2012). Angiotensin receptor blockade recovers hepatic UCP2 expression and aconitase and SDH activities and ameliorates hepatic oxidative damage in insulin resistant rats. *Endocrinology*.

[84] Andrews Z. B., Horvath T. L. (2009). Uncoupling protein-2 regulates lifespan in mice. *American Journal of Physiology—Endocrinology and Metabolism*.

[85] de Souza B. M., Michels M., Sortica D. A. (2015). Polymorphisms of the *UCP2* gene are associated with glomerular filtration rate in type 2 diabetic patients and with decreased *UCP2* gene expression in human kidney. *PLoS ONE*.

[86] Yoshida T., Kato K., Fujimaki T. (2009). Association of genetic variants with chronic kidney disease in Japanese individuals. *Clinical Journal of the American Society of Nephrology*.

[87] Tripathi G., Sharma R. K., Baburaj V. P., Sankhwar S. N., Jafar T., Agrawal S. (2008). Genetic risk factors for renal failure among north Indian ESRD patients. *Clinical Biochemistry*.

[88] Rubattu S., Di Castro S., Cotugno M. (2015). Protective effects of *Brassica oleracea* sprouts extract toward renal damage in high-salt-fed SHRSP: role of AMPK/PPAR*α*/UCP2 axis. *Journal of Hypertension*.

[89] Pagliaro B., Santolamazza C., Simonelli F., Rubattu S. (2015). Phytochemical compounds and protection from cardiovascular diseases: a state of the art. *BioMed Research International*.

[90] Aon M. A., Cortassa S., Juhaszova M., Sollott S. J. (2016). Mitochondrial health, the epigenome and healthspan. *Clinical Science*.

[91] Schapira A. H. (2006). Mitochondrial disease. *The Lancet*.

[92] Witasp A., Carrero J. J., Heimbürger O. (2011). Increased expression of pro-inflammatory genes in abdominal subcutaneous fat in advanced chronic kidney disease patients. *Journal of Internal Medicine*.

[93] Ye J., Li J., Xia R., Zhou M., Yu L. (2015). Prohibitin protects proximal tubule epithelial cells against oxidative injury through mitochondrial pathways. *Free Radical Research*.

[94] Kornfeld O. S., Hwang S., Disatnik M.-H., Chen C.-H., Qvit N., Mochly-Rosen D. (2015). Mitochondrial reactive oxygen species at the heart of the matter: new therapeutic approaches for cardiovascular diseases. *Circulation Research*.

